# Developmental stages of cultivated strawberry flowers in relation to chilling sensitivity

**DOI:** 10.1093/aobpla/plv012

**Published:** 2015-02-06

**Authors:** Maria Teresa Ariza, Carmen Soria, Elsa Martínez-Ferri

**Affiliations:** IFAPA, Centro de Churriana, Cortijo de la Cruz s/n, 29140 Churriana, Málaga, Spain

**Keywords:** Critical periods, flower development, flower differentiation, *Fragaria* × *ananassa*, low temperature, pistil, pollen

## Abstract

Environmental factors affecting flower development may limit the yields of fruiting crops worldwide. In temperate regions, chilling temperatures during flower development can compromise fruit production, but their negative effects vary depending on the differing susceptibilities of each developmental stage. The cultivated strawberry (*Fragaria*× *ananassa* Duch.) is widely grown worldwide but financial returns are influenced by sudden shifts to chilling temperatures occurring during the cropping cycle. Despite this important limitation, knowledge of *F.*× *ananassa* flower development is lacking, in contrast to the diploid wild-type strawberry (*F. vesca*). In this study we describe steps in floral development of cultivated strawberry and define their vulnerability to chilling temperatures. To achieve this, flower buds from strawberry plants of cv. ‘Camarosa’ were labelled and monitored from bud initiation until anthesis. Description of morphological and functional changes during flower development was based on histological sections and scanning electron microscopy. To determine the impact of low temperatures at different developmental stages, plants carrying buds of different sizes were chilled at 2 °C for 24 h. Several parameters related to male and female gametophyte development were later evaluated in flowers as they approached anthesis. *Fragaria*× *ananassa* flower development was divided into 16 stages according to landmark events. These stages were similar to those documented for *F. vesca* but three new additional intermediate stages were described. Timing of developmental processes was achieved by correlating developmental staging with specific bud sizes and days before anthesis. Time to reach anthesis from early bud stages was 17–18 days. During this period, we detected four critical periods vulnerable to low temperatures. These were mostly related to male gametophyte development but also to injury to female organs at late developmental stages. These results provide the essential groundwork on floral biology of cultivated strawberry that is a prerequisite for successful comparative studies of cold tolerance among genotypes during flower formation.

## Abstract (precis)

During strawberry flower development, a down-shift of temperatures below 2°C translates into injury to reproductive organs from early stages of bud development until the flower opens. Pollen development is especially vulnerable to chilling but carpels were also affected at the end of their maturation. By associating flower developmental stages to specific bud sizes, it was possible to identify when critical processes are taking place during flower development. These results can be used to shed light on susceptibility to low temperatures of other fruiting crops belonging to the *Rosaceae*.

## Introduction

Over 95 % of the Earth's surface experiences temperatures below 5 °C each year (Atkin *et al.* 1999). On clear days in winter and early spring, plants of extra-tropical regions are subjected to a combination of chilling temperatures and high solar irradiances. These conditions can often damage flower development of fruiting crops and, consequently, depress fruit production, since the capacity of a flower to become a fruit is established before anthesis (Rodrigo *et al*. 2009). Cool temperatures therefore have negative effects on fruit set by influencing processes taking place throughout flower development (Mercado *et al.* 1997; Ariza *et al*. 2011, 2012).

These effects can be especially prevalent in annual-fruiting crops such as strawberry that produce flowers throughout the cropping cycle. The commercial strawberry (*Fragaria*× *ananassa* Duch.) is grown from the tropics to the Arctic (Galletta and Bringhurst 1990), with main production areas in the USA (Salamé-Donoso *et al.* 2010), Mexico, Turkey (FAOSTAT 2014) and Spain (López-Medina *et al*. 2001; Dominguez *et al*. 2006). In the current growing areas, chilling temperatures are common during late winter and early spring, detrimentally affecting flower development and yields produced in the early season (from January to March; Ariza *et al.* 2011). Good financial returns are closely related to the production of high-quality yields, good flavour, appropriate fruit size and symmetric shape (Perkins-Veazie 1995), but also with the production of early yields (Rowley *et al.* 2010).

Strawberry flowers that develop in early winter are especially prone to chilling. Injury to the inflorescence, anthers and receptacle occurs at temperatures below 0 °C (Ki and Warmund 1992) and low temperatures 7 weeks before harvest have been related to misshapen fruit (Ariza *et al*. 2012). Low temperatures also affect the integrity of the floral structures (Ariza *et al*. 2011). The negative effects of cold on flower development and fruit production are thought to vary depending on the susceptibility of the processes associated with each floral developmental stage (Ohnishi *et al.* 2010). This is cultivar-dependent (Takeda *et al.* 2002) and depends on the range and duration of the chilling.

The suitability to different environments for cultivating strawberry depends on the cultivar; its genetic complexity giving rise to contrasting acclimation abilities in different environments. *Fragaria*× *ananassa* has therefore been termed a microclimatic crop (Rieger 2005). The genetic complexity of cultivated strawberry is greater than in *F. vesca*, and its octoploid nature gives rise to a multiplicity of flowering responses (Heide *et al.* 2013). It may also result in differences during flower development. Although floral development in the diploid *F. vesca* has been well studied (Hollender *et al*. 2012), there is less information for *F.*× *ananassa* and on the effects of chilling during flower differentiation, morphogenesis and maturation of its reproductive structures.

The aim of this study was to identify the main processes that comprise flower differentiation in *F.*× *ananassa* and to determine whether they are sensitive to chilling. We used cv. ‘Camarosa’ (University of California 1992; US Plant Patent #08708), a widely cropped early season short day cultivar, suitable for the major cropping areas. It is intended for use as a model for *F.*× *ananassa* flower development. Its adoption will help to detect which processes are potentially vulnerable to cold temperatures in other strawberry cultivars.

## Methods

### Plant material

Ninety strawberry plants of cv. ‘Camarosa’ (Larson 2001), from a commercial high-elevation nursery of Castille-Leon (northeastern Spain; lat. 41°30′N, long. 4°55′W, alt. 900 m above sea level), were planted into 6 L pots filled with a commercial substrate (Substrate Projar Professional; Fertilizer N-P-K: 14–16–18 + micronutrients, Projar^®^, Valencia, Spain) and sand (2 : 1, v/v) and, with an over-layer of Perlite. The plants were grown in a controlled temperature glasshouse (22 ± 2 °C and 70 ± 10 % RH) at the IFAPA Centro de Churriana in Málaga, Spain.

### Flower bud size and days to anthesis

To analyse the time to anthesis (days before anthesis), five primary flower buds from 0.1 to 1.4 cm length were labelled, dated and monitored daily until anthesis. The length of flower buds was determined each day using a digital calliper (Kern^®^) and 14 intervals of 1 mm each were linked to a particular floral stage. To characterize the flower, 30 primary flowers were selected 1 day before anthesis. The total number of pollen grains per flower, the percentage of viable and non-viable pollen, ovule viability and immature stigmas were estimated as described below.

### Flower development

To analyse the processes of development, three flower buds from each size category were preserved in FAA (5 : 5 : 90 v/v, formalin : acetic acid : 70 % ethanol; Johansen 1940) for embedding in paraffin (Paraplast, Merk) or preserved into GAA (glutaraldehide 2.5 % in 0.03 M phosphate buffer) for embedding in synthetic resins (Historesin, Technovit 7100, Microm). Samples were then sectioned with a rotary microtome (Jung supercut, 2065, Leica), stained and observed under stereoscope (Leica, MZ 95) or microscope (Leitz Laborlux 12). Photographs were taken with a digital camera (Canon PowerShot S70, 7.1 Megapixels) and analysed using an image processor program (UTHSCA, Imagen Tool 3.0).

Samples embedded in paraffin were sectioned at 10 μm and stained with toluidine blue or PAS (Feder and O'Brien 1968) for observation of the anther and pistil structure and with acetocarmine (40 % acetic acid saturated with carmine; Mercado *et al*. 1994) to examine pollen grains. At different buds sizes, 20 anthers and 10 carpels per region of each bud were analysed for the length of outer and inner anthers, length of the receptacle, diameter of pollen grain and length of apical and basal carpels in the receptacle.

For a functional approach, three floral buds 0.1, 0.4, 0.6, 1.1 and 1.3 cm long were included in synthetic resin and cut 2 μm thick. The tissues were then stained with acridine orange to detect cellular activity (i.e. synthesis of RNA and DNA; Dudley *et al*. 1987), with aniline blue to identify callose (Currier 1957; Linskens and Esser 1957) and with Auramine O to detect lipids (Heslop-Harrison 1977). Observations were made under a light microscope equipped with UV epifluorescence using a band pass 355–425 exciter filter and an LP 460 barrier filter.

Anthers, carpels and pollen were also observed by scanning electron microscopy (SEM; Jeol, JSM 840). Five flower buds of each category were submerged in 100 % ethanol after removing sepals and petals. The samples were dehydrated at critical point in liquid CO_2_ in a Balzers CPD-030. Dehydrated material was mounted on stubs and coated with gold in a Jeol, JFC 1100 sputter coater prior to SEM photography.

### Effect of chilling on floral development

After flowering initiation, 30 plants were transferred to a phytotron (IBERCEX, ASL S.A.) at 2 °C for 24 h with a photoperiod of 11 h, and then returned to the glasshouse. A batch of 30 plants kept in the glasshouse served as controls. Before chilling, all the flower buds were dated and monitored up to 2 days before anthesis. Five to 10 flowers per treatment at each bud stage were observed under a microscope at the same time.

To evaluate the effect of chilling on male structures, the total number of pollen grains per flower, percentage of germinated pollen grains and percentage of non-viable pollen were recorded in control and chilled plants. Anthers from 5 to 10 flowers per treatment were placed in Petri dishes inside a sealed dry chambers containing silica gel for 24 h to promote release of pollen grains. The pollen was placed in 1.5 mL vials and 150 μL of a germination medium consisting of 10 % glucose and 150 ppm boric acid added (Ariza *et al*. 2006). Two aliquots of the solution were separated to estimate the number of pollen grains and to assess pollen viability. To estimate the number of pollen grains per flower, pollen stained with 1 % acetocarmine was counted using a Neubauer chamber under an optical microscope (Leitz, Laborlux 12). Viability of the pollen at each date was assessed by incubating vials for 4 h at room temperature under light conditions and continuous shaking (Hortynski and Zebrowska 1991) before staining with 1.0 % acetocarmine. Germination *in vitro* was examined for ∼200 pollen grains by light microscopy (Leitz, Laborlux 12, Germany; Fig. 2E). Pollen was considered viable and fertile when the length of the tube was greater than or equal to the diameter of the grain (Mercado *et al*. 1994). The percentage of germinated pollen was estimated on the basis of the number of stained pollen grains (undisrupted and potentially viable). The percentage of non-viable pollen was calculated from the number of non-stained pollen grains.

To analyse the effect of the chilling on ovule viability and development of the stigmas, 20 carpels per flower were squashed and stained with aniline blue for observation by light microscopy. Carpels were separated into tubular- (Type I), heart- (Type II) and scalloped-shaped (Type III) (Ariza *et al*. 2011) and the percentage of immature stigmas calculated. Carpels with Type I and II stigmas were considered immature, and Type III stigmas were mature and receptive (Ariza *et al*. 2011). An ovule was considered aborted if it stained with aniline blue (Anvari and Stösser 1978; Ariza *et al*. 2011).

### Statistical analysis

Statistical analyses were performed using the analytical software STATISTIX 9.0 (Analytical Software, Tallahassee, FL, USA). To test the relationship between days before anthesis and flower bud sizes, length of anthers, diameter of pollen grain and length of carpels, lineal regression models were used. To evaluate the effect of chilling on the percentage of germinated pollen grains, percentage of non-viable pollen, number of pollen grains, type of stigma and percentage of aborted ovules, data of these variables from flowers at the same bud stage when chilling were subjected to a one-way ANOVA (*n* = 5–10 flowers per date). Prior to ANOVA, percentage data were transformed by arcsine and normality and homogeneity assumptions were tested by Kolmogorov–Smirnov and Cochran's *C* tests. Data were back-transformed to give the percentages of decrease or the fold increase.

## Results and Discussion

### The morphology and development of the flower in *F.*× *ananassa*

At anthesis, *F.*× *ananassa* flowers have a two whorl calyx (inner and outer) of 12.8 ± 0.2 alternating sepals. A whorl of 5.3 ± 0.1 white petals is present interior to the sepals. Subsequently, two whorls (inner and outer) of 22.8 ± 0.3 stamens form and surround the central receptacle dome. Each flower carries 248 384 ± 12 469 pollen grains; of which 47.9 ± 1.9 % are viable and 13.7 ± 0.7 % are non-viable. The pistil of each flower is the innermost whorl made up of 326 ± 18 carpels. Each carpel consists of a stigma, a style an ovary and one ovule (Hancock 1999). Carpels are embedded in the epidermis of the receptacle in a spiral pattern. The percentage of immature stigmas and aborted ovules was 7.7 ± 1.4 % and 12.3 ± 1.2 %, respectively. These results highlight a degree of functional failure of the reproductive structures in *F.*× *ananassa* under non-stressful conditions. However, these modest levels of failure do not necessarily translate into reduce fruit set or damage to development (Ariza *et al*. 2011, 2012).

**Figure 1.**
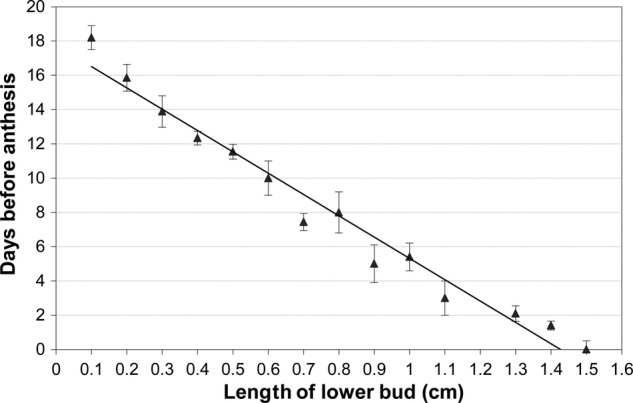
Relationship between days before anthesis and length of flower bud in cultivated strawberry. The regression model fitted (days before anthesis = −12.443*x* + 17.754, *R*^2^ = 0.97, *P* < 0.001) is depicted. Each data point is the mean ± SE, *n* = 20.

The flower of *F.*× *ananassa* has considerably more bracts per whorl and is bigger than *F. vesca* (∼1.4 cm versus ∼0.7 cm, respectively; Hollender *et al*. 2012). In *F.*× *ananassa*, flower buds increased linearly from 0.1 to 1.5 cm at anthesis over 17–18 days with a growth rate ∼1 mm day^−1^ (Fig. 1). Time to reach anthesis may differ between both species but data for *F. vesca* are not available. Changes in bud growth were linked to landmark events, allowing a chronological ranking of various developmental stages.

**Figure 2.**
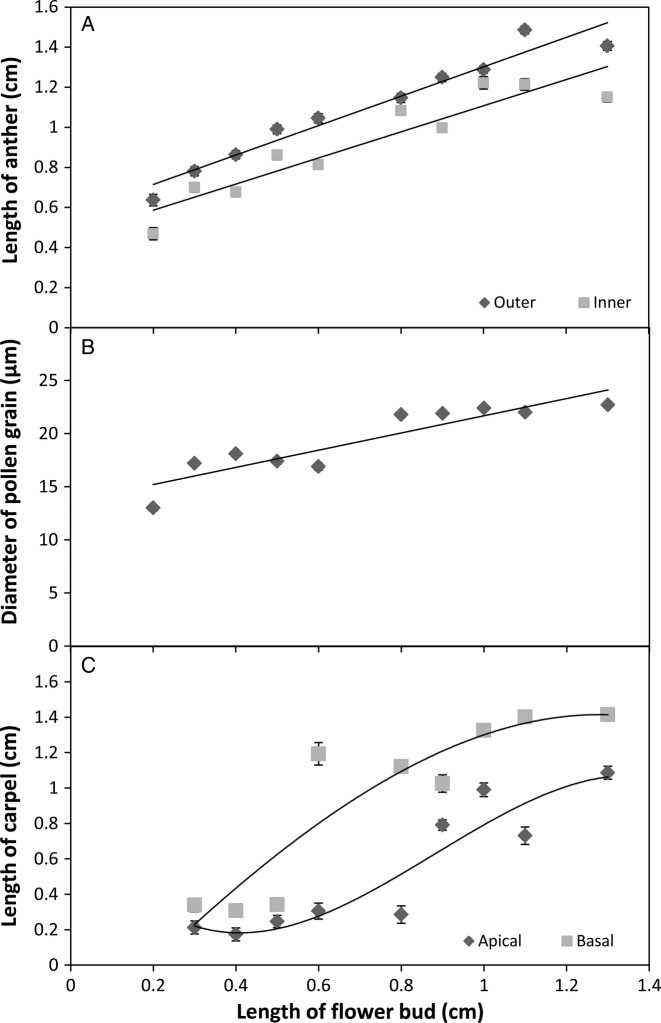
Relationship between the length of flower bud and length of outers and inners anthers (A), diameter of pollen grain (B) and length of carpels in apical and basal region of the receptacle (C). Each data point is the mean ± SE, *n* = 9–45. Fitting models of regression depicted are: length of outer anthers = 0.734*x* + 0.5679, *R*^2^ = 0.95; Length of inner anthers = 0.6513*x* + 0.456, *R*^2^ = 0.87; Pollen grain diameter = 8.0943*x* + 13.593, *R*^2^ = 0.82; Length of apical carpels = −2.1203*x*^3^ + 5.6*x*^2^ − 3.5177*x* + 0.8289, *R*^2^ = 0.86; Length of basal carpels = −0.3122*x*^3^ − 0.3387*x*^2^ + 2.4045*x* − 0.4533, *R*^2^ = 0.84.

There was a linear relationship between days before anthesis and length of the anthers (Fig. 2A) and diameter of the pollen grain (Fig. 2B); in contrast, the relationship with carpel development was sigmoidal (Fig. 2C). Anthers from the outer part of the receptacle were larger than those from the inner part (Fig. 2A) indicating a maturity gradient from outside to inside. Similarly, basal carpels were longer than the apical ones (Fig. 2C), suggesting acropetal pistil development (Weberling 1989). These differences in the maturation of the reproductive structures within the same flower could be concomitant with different stages of gametophyte development (i.e. male and female) at a specific bud size. However, the model of floral development presented here for *F.*× *ananassa* integrates the main and significant changes occurring in the overall parts of the flower at a given time.

### Male gametophyte development

**Figure 3.**
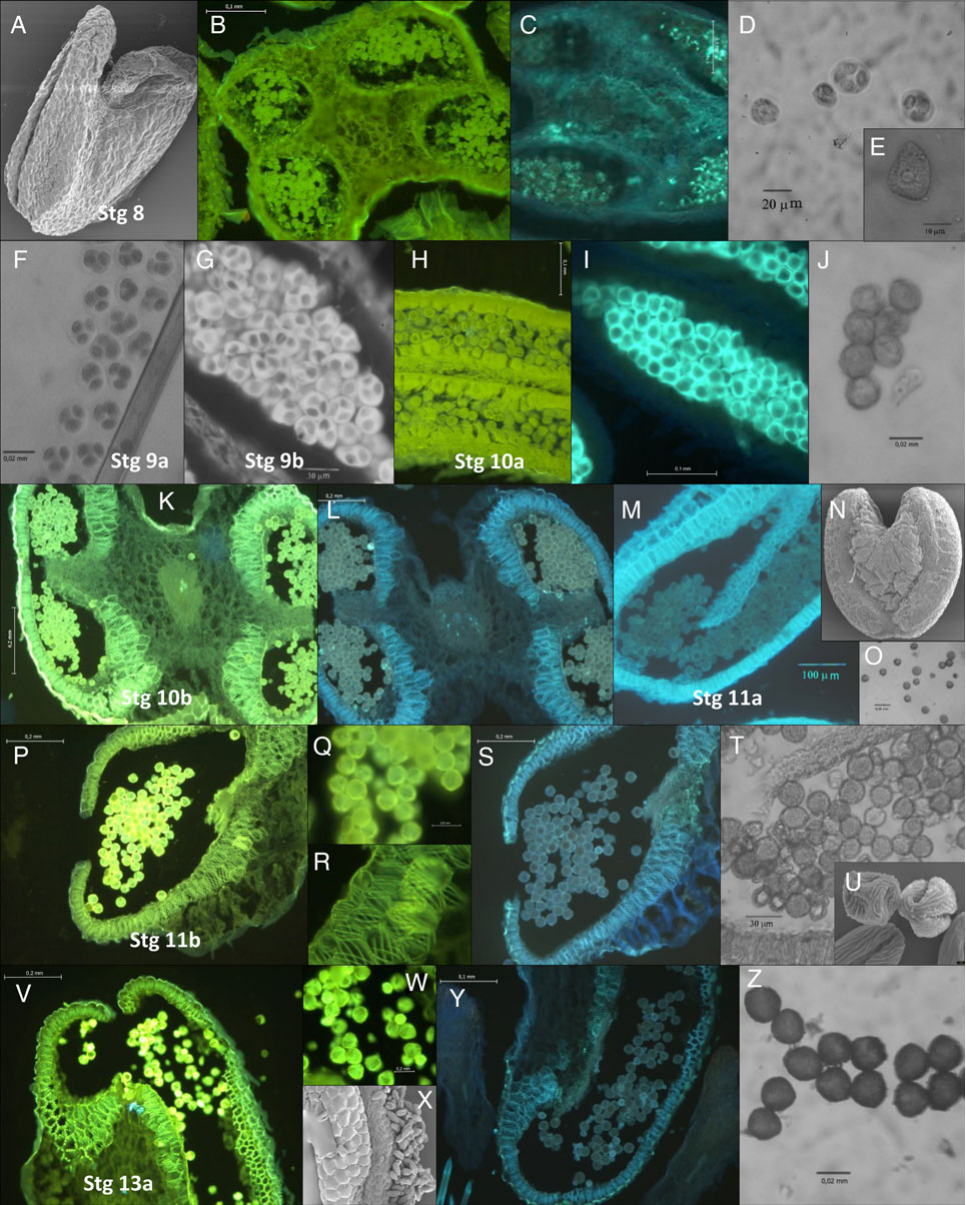
Micrographs of anther and pollen development of *F.*× *ananassa* flower buds at different developmental stages (Stg): stage 8 (A–E), stage 9a (F), stage 9b (G), stage 10a (H–J), stage 10b (K and L), stage 11a (M–O), stage 11b (P–U) and stage 13a (V–Z). Histological sections embedded in historesin are stained with Auramine O (green coloured) and aniline blue (blue coloured). Non-coloured pollen photographs are directly observed after squashing. Three-dimensional images of anthers and pollen were acquired by scanning electron microscopy.

At early stages of male gametophyte development (<1 cm bud size), a group of cells within the pollen sacs differentiate into archesporial cells. Similarly, primary parietal cells (PPCs) differentiate by mitotic division into sporogenous cells (PSCs; Stanley and Linskens 1974; Strasburger *et al.* 1994). Primary parietal cells give rise to endothecium, the middle layer of the anther and the tapetum (Yang *et al.* 1999), and PSCs differentiate into the pollen mother cells (PMCs) (Lord *et al*. 1989). At stage 8 (0.1 cm bud size), 18–17 days before anthesis, anthers are four-lobed, with walls differentiated into layers and PMCs were observed inside the pollen sacs (Fig. 3A–C). Pollen mother cells were negative to Auramine O, and positive to aniline blue indicating the presence of external callose (Fig. 3B and C, respectively). Also, angular cells were seen in the anthers (Fig. 3E), which might correspond to the tapetum cells aroused from PPCs at previous stages (stage 7, <0.1 cm; Lord *et al*. 1989). At this stage, PMCs began meiosis (Fig. 3D) which lasted until stage 9b (0.3 cm, 14 days before anthesis), when tetrads of four haploid microspores were observed within the locules (Fig. 3F and G). At stage 10a (0.4 cm, 13–12 days before anthesis), single rounded haploid microspores, still with a callose external layer (positive to aniline blue; Fig. 3E), are loose within the locule (Fig. 3H–J), indicating that microsporogenesis and meiotic reduction was completed at the earlier stage (stage 9b; 18–14 days before anthesis). This is in agreement with studies of bell pepper (Erickson and Markhart 2002) although it seems that timing of meiotic division is species dependent (Patterson *et al*. 1987; Porch and Jahn 2001). After meiosis, subsequent microgametogenesis and maturation of microspores takes place (Anger and Weber 2006).

At stage 10b (0.5–0.6 cm, 11–10 days before anthesis), pollen grains were round but only stained with Auramine O (Fig. 3K and L), indicating an outer layer of lipids (i.e. the exine). Secretion of the exine precursors is mediated by tapetum cells (Pacini and Juniper 1984; Scott *et al.* 2004; Yang *et al.* 2007) which also nourish the microspores during microspore maturation (Pacini 1990). These lipids contribute to the ornamentation of the pollen wall. There were also three apertures (i.e. the colpos) along the grain (Fig. 3T and U). Also, at this stage, fibrous bands on the epidermis and endothecium cells of the anther walls become evident (Fig. 3K and L). This may indicate initiation of tapetum degradation (Hollender *et al*. 2012). These cells continue to grow (Fig. 3M, P, R and S).

At stage 11a (0.7–0.9 cm, 9–6 days before anthesis), the final size of pollen grain was reached (Fig. 3Q and T). This is a time when the tapetum becomes fully degraded (Hollender *et al*. 2012) as a consequence of programmed cell death occurring at later stages of pollen development (Kawanabe *et al*. 2006).

At stage 11b (1.0–1.2 cm, 5–3 days before anthesis) pollen mitotic division occurs that creates the vegetative and generative nuclei. This observation agrees with that of Hollender *et al*. (2012) and with our later results on critical periods of susceptibility to low temperature discussed below. Anther dehiscence takes place at stage 13a (1.3 cm) and 1–2 days before anthesis. This occurs when flower buds are 1.4–1.5 cm long (stage 13b).

### Pistil and female gametophyte development

**Figure 4.**
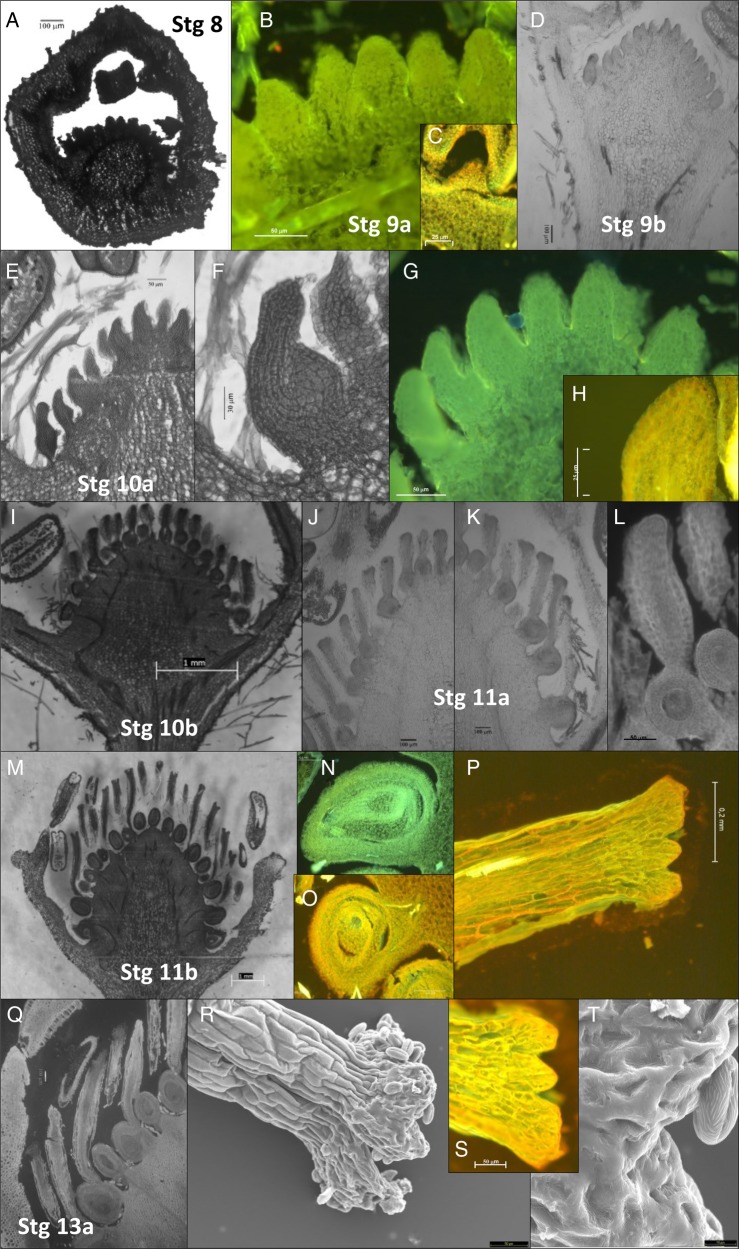
Micrographs of pistil development of *F.*× *ananassa* flower buds at different developmental stages (Stg): stage 8 (A), stage 9a (B and C), stage 9b (D), stage 10a (E–H), stage 10b (I), stage 11a (J–L), stage 11b (M–P) and stage 13a (Q–T). Histological sections embedded in paraffin were stained with PAS or toluidine blue (shown in black and white) for structural examination. Samples embedded in historesin were stained with auramine O (green coloured) and acridine orange (yellow coloured) for a morpho-functional approach. Three-dimensional images of stigmas were acquired by scanning electron microscopy.

At stages 8 and 9 (0.1 and 0.2 cm), the flower receptacle was fully covered by the protruding carpel primordia (Fig. 4A–C). The external lipid layer on the carpel surface and the considerable DNA and RNA synthesis observed in basal and apical carpels from early developmental stages (Fig. 4) point to changes in carpel size and development being closely related to active metabolic processes involved in rapid cell growth and differentiation (Hollender *et al*. 2012). From stages 9 (0.2 cm) to 9b (0.3 cm) carpel primordia undergo rapid morphological changes in 3 days (between 14 and 16 days before anthesis). This results in a transformation from thumb-like protuberances (Fig. 4A–C) to a bowling pin-shaped carpels at the receptacle base (Fig. 4D and E). As described in *F. vesca*, this rapid development involves the differentiation of epidermal carpel cells into several layers and the folding inward of ovary wall margins to envelop the single ovule containing the megasporocyte (megaspore mother cell: MMC) (Hollender *et al*. 2012). This differentiation in *F.*× *ananassa* was clear at stage 10a (13–12 days before anthesis) when the standard bottle-shape carpel morphology with anacrostily (Weberling 1989) was observed (Fig. 4F). At this stage, meiosis of MMC is probably occurring as reported for *F. vesca* (Hollender *et al*. 2012).

From stage 10b (0.5–0.6 cm, 11–10 days before anthesis) to stage 11a (0.7–0.9 cm, 9–6 days before anthesis), rapid elongation of the style, accompanied by ovary expansion, takes place and carpels shaped like a music-note can be observed at the receptacle base (Fig. 4I–L). This is associated with the prevalence of DNA synthesis.

At stage 11b (1.0–1.2 cm, 5–3 days before anthesis) the ‘music-note’ carpels reach their maximum size (Fig. 4M), the ovary is completely formed (Fig. 4N and O) and the stigmas become scalloped (Fig. 4P). The three consecutive mitosis of the chalazal megaspore may then generate the embryo sac (Souza *et al*. 2002; Yang 2005). This is in accordance with Hollender *et al*. (2012) and with the observed vulnerability of this stage to low temperatures (see below).

Finally, at stage 13a (1.3 cm, 2–1 days before anthesis), mature and receptive scalloped-shaped stigma were observed (Ariza *et al*. 2009, 2011). These stigmas displayed a high metabolic activity, which is consistent with observed lipid secretion on the stigma surface (Fig. 4R–T) which aids adhesion of the pollen (Avigdori-Avidov 1986). These findings suggest that at 1 or 2 days pre-anthesis flower reproductive structures are fully functional in cultivated strawberry.

### Effect of chilling on floral development: critical periods

**Figure 5.**
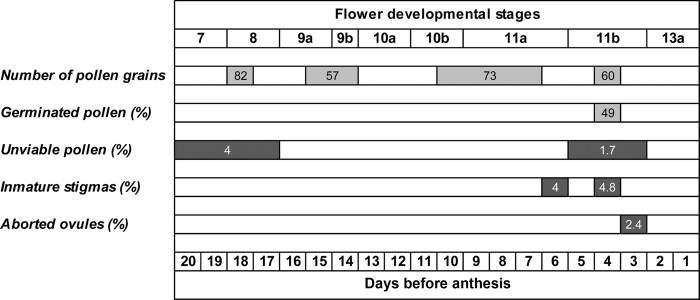
Low-temperature critical periods (grey bands) in days before anthesis of strawberry flower development, detected after subjecting flowers buds at different developmental stages to chilling (24 h at 2 °C). Periods were identified when strawberry flowers at stage 13a displayed significant (*P* < 0.05; *n* = 5–10) differences between the cold and control treatments in any of the following variables: number of pollen grains, percentage of germinated pollen, percentage of unviable pollen, percentage of immature stigmas and percentage of aborted ovules. The *n*-fold increase or the decrease percentages for each variable are depicted in the dark grey bands and light grey bands, respectively.

In flowering plants, the gametophyte phase is known to be sensitive to hot or cold, i.e. freezing and chilling temperatures even for a short period (Zinn *et al*. 2010; Parish *et al*. 2012). In the present study four critically sensitive periods of flower development in *F.*× *ananassa* were identified (Fig. 5) by subjecting flower buds at different stages to chilling.

The earliest stage showing susceptibility to low temperatures is male gametophyte development at stage 8 or before (20–17 days before anthesis). This was revealed as a significant decrease (up to 82 %) in the number of pollen grains, and by a 4-fold increase of the percentage of non-viable pollen (Fig. 5). These results demonstrate that temperature <2 °C negatively affect PMC development and anthers differentiation on endothecium, middle layer and tapetum, respectively.

The second stage showing chilling susceptibility was detected 15–14 days before anthesis (stages 9a and 9b). Here a decrease in the number of pollen grains per flower by up to 57 % was observed. This effect has been previously associated with disruption of PMCs meiosis under high temperatures (Kim *et al*. 2001; Erickson and Markhart 2002). Likewise, our results reveal that meiosis of PMCs in cultivated strawberry is also sensitive to low temperature. This is in agreement with recent studies reporting signs of cytomixis of PMCs at prophase I, contributing to the death of PMCs, as a consequence of chilling (Barton *et al*. 2014).

A third chilling sensitive stage occurs 10–7 days before anthesis (stages 10b and 11a). Dame here decreased the number of pollen grains by 73 %, indicating that microspore development and tapetum degeneration are affected (Parish *et al*. 2012). Pollen sterility induced by cold might be due to a disruption of sugar metabolism in the tapetum, ultimately abolishing starch accumulation (i.e. energy reserves) and exine secretion by the pollen grains (Oliver *et al*. 2005).

Finally, chilling 5–3 days before anthesis (stage 11b) decreased the percentage of germinated pollen grains by 49 % and increased percentage of non-viable pollen by 1.7-fold. This indicates that microspore mitotic processes are being disrupted (Satake and Hayase 1970), an effect probably associated with microtubules disassembly during mitosis. Microtubules are known to be highly thermosensitive (Hepler and Hush 1996). On the other hand, chilling decreased the number of pollen grains by 60 %. This may be a result of physical disruption of pollen grains and of cell death induced by deleterious effect of low temperatures (Zinn *et al*. 2010).

Female reproductive organs were only affected by chilling 3–6 days before anthesis (stages 11a to 11b) when there was up to a 2.4- and 4.8-fold increase in the percentage of aborted ovules or of immature stigmas, respectively (Fig. 5). This suggests that low temperatures damage embryo sac formation and delays stigmas maturation. Both these phases are thus cold sensitive. This agrees with Ebadi *et al*. (1995) who reported a decline in ovule viability by low temperatures before anthesis. In contrast, other reports showed that low temperatures did not affect ovule and stigma receptivity in strawberry (Ariza *et al*. 2011). These discrepancies must be related to differences in the range of chilling temperatures tested by Ariza *et al*. (2011) and in the present study (>4 and 2 °C, respectively). This suggests that female reproductive structures and esporogenesis of cultivated strawberry are fairly tolerant to temperatures >2 °C. These findings underline the lower sensitivity of female reproductive processes to cold temperatures (Dupuis and Dumas 1990; Saini and Lalonde 1997). The chilling sensitivity of other strawberry cultivars now needs to be addressed since responses to environmental stress are often cultivar-related (Ledesma *et al.* 2007).

## Conclusions

The present study describes the morpho-functional processes of flower development in cultivated strawberry (*F.*× *ananassa*) and reveals which steps are susceptible to damage from chilling at 2 °C. It identifies a close relationship between flower developmental stages and the size of the flower bud as it develops over ∼18 days. Flower developmental stages in *F.*× *ananassa* were defined by macroscopic and microscopic morpho-functional changes in the reproductive structures as flower bud size increased. Most of these changes reflect those described recently for *F. vesca* during flower differentiation (Hollender *et al*. 2012), but additional stages were identified in *F.*× *ananassa* buds of intermediate sizes (Table 1).

**Table 1.** Stages of flower development and key events in pollen and carpel development of cultivated (*F.*× *ananassa*) and wild strawberry (*F. vesca*; Hollender *et al*. 2012). Italics denote those events that are inferred from *F. vesca*. The asterisk indicates those events vulnerable to chilling injury. ^1^Described in stage 12 on *F. vesca*.

*F. vesca* floral stage	*F.*× *ananassa* floral stage	*F.*× *ananassa* bud length (cm)	Days before anthesis	Pollen	Fig.	Carpel	Fig.
*7*	7	<0.1	>18	*Endothecium, middle layer and tapetum arise from parietal cells. PMCs appear.*		*Round carpel primordia reach the receptacle apex. No cell differentiation of epidermal layer.*	
*8*	8	0.1	18–17	*PMCs enter in meiosis. Tapetum cells present. Four anther locules are clearly distinct.	3A–E	Receptacle fully covered with carpels.	4A
*9*	9a	0.2	16–15	*PMCs in meiosis. Tetrads confined and imbibed in callose.	3F	Thumb-like carpel primordia at the receptacle apex.	4B and C
	9b	0.3	14	Meiosis is complete and callose wall holding tetrads together start to disintegrate.	3G	Bowling pin-shaped carpel primordia at the receptacle base. *Indented carpel walls* and *MMC might be visible.*	4D
*10*	10a	0.4	13–12	Round microspores are loose in the locule surrounded by a callose layer. Complete tetrad disintegration.	3H–J	Central carpel constriction divide organ into two almost equal apical and basal parts. MMC might enter in meiosis.	4E and F
	10b	0.5–0.6	11–10	*Microspore exine wall develops. Tapetum degeneration is initiated.	3K and L	Rapid elongation of style and ovary expansion.	4I
*11*	11a	0.7–0.9	9–6	*Tapetum is fully degraded and endothecium cells increase in size. Pollen final size reached.	3M–O	Idem. Music-note like carpels at the receptacle base.	4J–L
	11b	1–1.2	5–3	*Pollen mitotic division occurs (not obvious, deduced by chilling at 2 °C)	3P–U	*Fully formed embryo sac. Final size of music-note like carpels.^1^ Scalloped stigmas with high metabolic activity.	4M–P
*13*	13a	1.3	2–1	Anther dehiscence	3V–Z	Scalloped and mature stigmas with moist secretion for pollen adhesion.	4Q–T
	13b	1.4–1.5	0	Anthesis			

In addition to contributing to the knowledge of the floral biology of Rosaceae, and strawberry, the present study implicates particular developmental processes as potentially sensitive to low temperature. The findings will also aid future comparative studies of species or cultivars aimed at identifying differences in tolerance and susceptibility. Such work will benefit breeders and growers working in areas where sudden down-shifts of temperature take place during the cropping cycle, especially during flowering.

## Sources of Funding

This research was funded by the Spanish Instituto Nacional de Investigación Agraria (INIA) RTA2004-008 and CC10-009-C02-02 projects, and co-financed by European FEDER funds. M.T.A. was supported by a research grant financed by the Spanish INIA and is currently supported by IFAPA. Junta de Andalucia (20 %) and by the Programa Operativo Fondo Social Europeo (FSE) de Andalucía 2007–2013 (80 %) under the topic ‘*Andalucía se mueve con Europa*’.

## Contributions by the Authors

All authors were involved in designing the experiments of this research, sampling, data handling, statistical analysis and manuscript preparation and submission. Microscopy technical work was mainly done by M.T.A.

## Conflicts of Interest Statement

None declared.
